# Prevalence of Depression in Coronary Artery Bypass Surgery: A Systematic Review and Meta-Analysis

**DOI:** 10.3390/jcm9040909

**Published:** 2020-03-26

**Authors:** María Correa-Rodríguez, Moath Abu Ejheisheh, Nora Suleiman-Martos, María José Membrive-Jiménez, Almudena Velando-Soriano, Jacqueline Schmidt-RioValle, José Luis Gómez-Urquiza

**Affiliations:** 1 Faculty of Health Sciences, University of Granada, Avenida de la Ilustración N. 60, 18016 Granada, Spain; macoro@ugr.es (M.C.-R.); moad.ibrahem@hotmail.com (M.A.E.); jschmidt@ugr.es (J.S.-R.);; 2 Instituto de Investigación Biosanitaria, IBS, 18012 Granada, Spain; 3 Faculty of Health Sciences, University of Granada, Campus Universitario de Ceuta, C/Cortadura del Valle s/n, 51001 Ceuta, Spain; 4 University Hospital of Ceuta. Institute of Health Management. C/Colmenar s/n, 51003 Ceuta, Spain; mariajose.membrive@gmail.com; 5 University Hospital Virgen de las Nieves. Andalusian Health Service. Av. de las Fuerzas Armadas 2, 18014 Granada, Spain; srtavelando@correo.ugr.es

**Keywords:** coronary artery bypass graft, depression, mental health, meta-analysis, prevalence, surgery, systematic review

## Abstract

Coronary artery bypass graft surgery (CABG) might adversely affect the health status of the patients, producing cognitive deterioration, with depression being the most common symptom. The aim of this study is to analyse the prevalence of depression in patients before and after coronary artery bypass surgery. A systematic review and meta-analysis was carried out, involving a study of the past 10 years of the following databases: CINAHL, LILACS, MEDLINE, PsycINFO, SciELO, Scopus, and Web of Science. The total sample comprised *n* = 16,501 patients. The total number of items was *n* = 65, with *n* = 29 included in the meta-analysis. Based on the different measurement tools used, the prevalence of depression pre-CABG ranges from 19–37%, and post-CABG from 15–33%. There is a considerable presence of depression in this type of patient, but this varies according to the measurement tool used and the quality of the study. Systematically detecting depression prior to cardiac surgery could identify patients at potential risk.

## 1. Introduction

Coronary artery disease (CAD) is one of the leading causes of death in developed countries, and it is associated with deteriorated quality of life, disability, and premature death [1]. The usual surgical treatment involves coronary artery bypass graft surgery (CABG). This technique is based on revascularisation by diverting blood flow to other arteries to increase the blood supply to the heart muscle [2].

Although CABG surgery increases life expectancy [3], it is associated with multiple physical complications, including myocardial infarction, stroke, and even kidney failure [4]; in addition to psychological consequences, such as mood disorders, fatigue, weakness, stress, anxiety, and depression [5].

Short-term recovery factors include a longer hospital stay, pain, and infection, which may predispose towards cognitive disorders, like anxiety and depression [6]. In the long term, it is estimated that at least 25% of patients will experience deteriorated quality of life after a CABG; and, it even doubles the post-surgery risk of future cardiac events and mortality related to high levels of anxiety and depression [7].

In particular, depression is considered to be one of the main reasons for reduced well-being, having a negative impact on a patient’s quality of life, as well as their social and family life. It is a strong risk factor for mortality, being related to the occurrence of new cardiac events and reduced functionality up to six months post-CABG surgery, increasing the risk of hospital readmission in up to 20% of patients, due to complications including infection, arrhythmia, and volume overload [8].

Diagnosis is sometimes difficult, since symptoms, such as loss of appetite, sleep cycle disturbance, and constant fatigue, may be superimposed over the same symptoms that were derived from surgery. For this reason, determining the degree to which a CABG can affect a patient’s mental, psychological, and social skills, and, specifically, analysing the level of depression, requires the use of multiple tools validated during a clinical interview [9,10].

A number of factors seem to influence the relationship between depression and CABG, including biological alterations (cardiac rhythm alterations, tone of cardiac muscle, hormone levels, and reduced brain perfusion) [11]. However, in many cases, the high prevalence of mood disorders cannot be explained by the severity of the illness, but is instead related to psychosocial factors, such as socioeconomic status, lifestyle (adherence to the recommended diet or prescribed treatment), or the level of social support [12].

Even though the effects of CABG have been studied in terms of morbidity, mortality, and organ function, the effect or influence it has on mood disorders, like depression, remains unclear. It seems that depression predicts how much a patient’s health will deteriorate. Therefore, it is extremely important to assess how the disease affects a patient, as this can influence the therapeutic benefit and, consequently, which interventions and care are prioritised, and which self-care strategies are implemented both before and after surgery [13,14].

Although depression is considered to have a negative impact on patient recovery, few studies have examined the association between CABG and depression. Some systematic reviews have analysed the risk factors [15], and there are also reports regarding the effect of certain interventions [16,17]. However, to our knowledge, no meta-analysis studies that include a prevalence analysis have been exclusively undertaken on CABG patients.

Describing the levels of depression in CABG patients is essential for analysing the importance of this surgery with regard to depression levels. The purpose of this work is, therefore: (1) to analyse the prevalence of depression in patients both before and after CABG surgery; and, (2) to analyse the depression levels over time.

## 2. Materials and Methods

The data were extracted and analysed based on the recommendations of preferred reporting items for systematic review and meta-analysis (PRISMA) 2015 [18].

### 2.1. Search Strategy

A search was conducted of CINAHL, LILACS, MEDLINE, PsychINFO, SciELO, Scopus, and Web of Science in January 2020. MeSH descriptors were used, with the search strategy being: “(depression OR depressive disorder) AND (coronary artery bypass grafting)”.

### 2.2. Inclusion and Exclusion Criteria

The inclusion criteria were the following: (1) full text of quantitative primary studies; (2) men and women aged over 18; (3) no psychiatric pathology or illness; (4) CABG surgery; (5) study of depression levels prior to or after CABG; (6) the use of a validated scale; (7) written in English, Portuguese, Spanish, or French; and, (8) published in the last 10 years.

The exclusion criteria were the following: (1) paediatric population; (2) a different type of cardiac surgery that was not exclusively CABG (CABG with valve replacement); (3) measurement of depression in relatives; (4) patients with an active treatment deriving from a psychiatric disorder; (5) data from duplicate articles in previous studies; and, (6) no depression data extracted using a validated scale.

### 2.3. Selection of Articles and Information Analysis

Firstly, two authors checked the title and abstract, and, secondly, the full text of the article. A third author was consulted in the case of discrepancy.

For the meta-analysis, we selected the data from those studies that used the same measurement tool, since the inclusion of several measurement tools would not permit the results to be integrated, due to different scores.

### 2.4. Data Extraction

The following variables were recorded: (1) data on the study (author, year, country); (2) type of CABG (first time, elective or emergency); (3) study characteristics (sample, type of study, sex, and follow-up time); (4) measurement tool; and, (5) mean, standard deviation, prevalence of depression. For clinical trials or quasi-experimental studies, we selected only the levels of depression prior to the programme intervention (baseline) or those relating to the control group.

We used the intraclass correlation coefficient to analyse coding reliability, obtaining an average value of 0.97 (minimum = 0.93; maximum = 1), and the Cohen’s kappa coefficient with a mean value of 0.94 (minimum = 0.92; maximum = 1).

### 2.5. Assessment of Quality and Measurement of Bias

Two independently authors assessed the quality of the studies, consulting with a third party in the event of a disagreement.

For observational studies (cohort and cross-sectional), we followed the guidelines in “Strengthening the Reporting of Observational Studies in Epidemiology” (STROBE) [19]. We followed the standards in the Cochrane Collaboration Risk of Bias tool for clinical trials [20].

We used a second quality assessment tool to analyse the level of evidence in accordance with the recommendations of the Oxford Centre for Evidence-Based Medicine [21] (Table 1).

### 2.6. Data Synthesis and Statistical Analysis

The meta-analysis included those studies that used the same tool for measuring depression. We performed six meta-analyses using a random-effects model and two meta-analyses using a fixed-effect model, for prevalence levels and confidence intervals, through the statistical package StatsDirect (version 3, StatsDirect Ltd., Cambridge, UK).

We used I^2^ to analyse the heterogeneity, grouping values into low (25%), moderate (50%), or high (75%) heterogeneity [22]. The publication bias was assessed using Egger’s test.

## 3. Results

**Figure 1:**
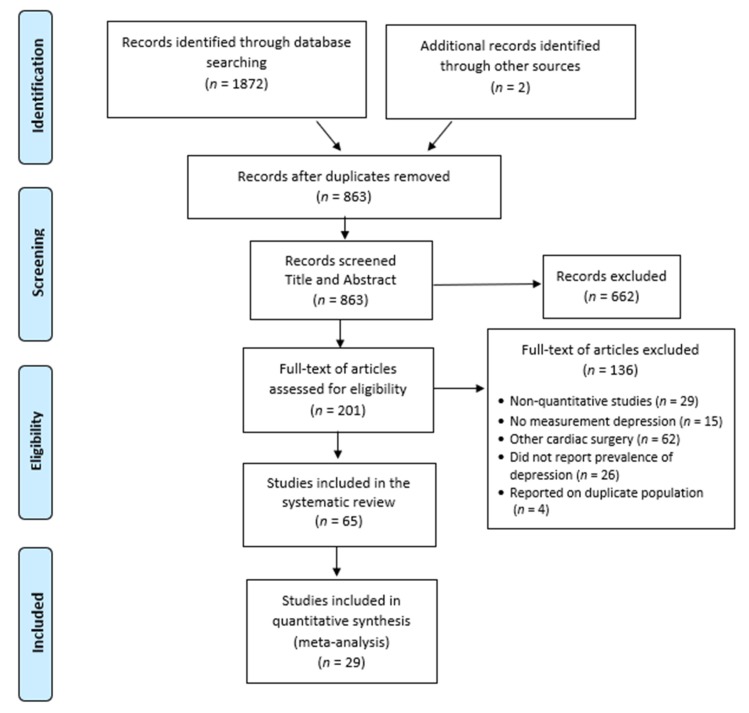
Preferred reporting items for systematic review and meta-analysis (PRISMA) flow-chart of included studies.

The search yielded a total of *n* = 1874 articles. After reading the title and abstract, 662 were excluded. Figure 1 shows the study selection process.

### 3.1. Characteristics of Included Studies

The total sample comprised 16,501 patients, predominantly male (*n* = 54). Most of the studies were cohort studies (*n* = 34), followed by cross-sectional studies (*n* = 12). Thirteen studies evaluated the levels prior to surgery, 23 after surgery, and 29 both before and after. Most of the studies were carried out in the USA (*n* = 17), followed by Germany (*n* = 7), Iran (*n* = 7), and Australia (*n* = 6) (Table 1). The depression follow-up ranged from a month prior to surgery (since the pre-assessment clinic appointment) [23] up to six years after surgery [24,25].

### 3.2. Measurement of Depression

We used a total of 15 measurement tools. The Hospital Anxiety and Depression Scale (HADS) (*n* = 18), Beck Depression Inventory (BDI) (*n* = 17), nine-item Patient Health Questionnaire (PHQ-9) (*n* = 9), and Centre for Epidemiological Studies Depression Scale (CES-D) (*n* = 4) were the measurement tools used (Table 1 and Supplementary Table S1).

### 3.3. Meta-Analysis

A total of 1217 patients were included in the meta-analysis prior to CABG surgery, and 596 patients after the operation. Egger’s test showed no publication bias in any case.

**Figure 2:**
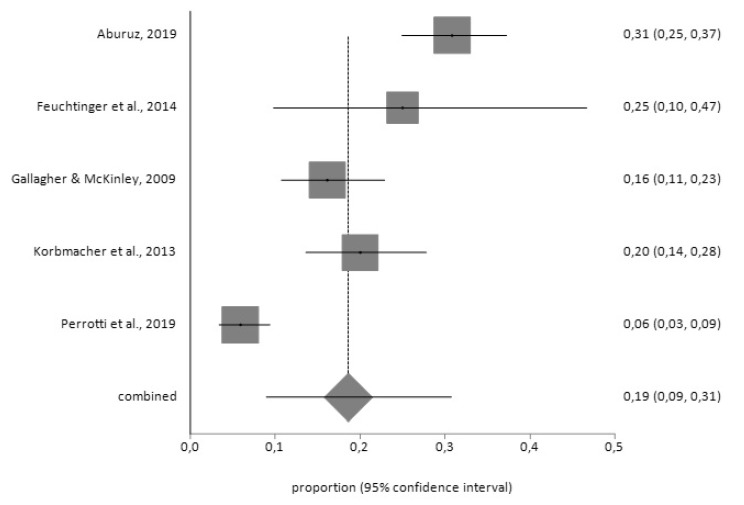
Forest plot for pre-CABG depression using Hospital Anxiety and Depression Scale (HADS).

**Figure 3:**
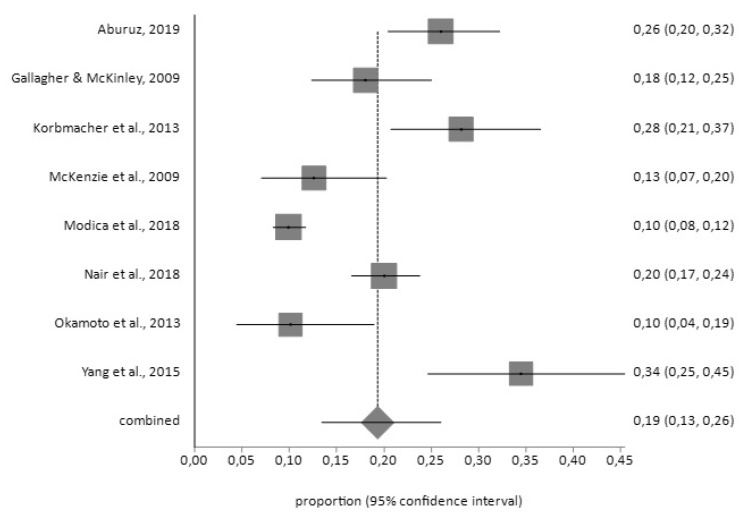
Forest plot for post-CABG depression using HADS.

For the HADS tool, the prevalence of depression prior to surgery (*n* = 144) was 19% (95% CI = 9–31) with a high degree of heterogeneity (I^2^ = 93.4%), while the prevalence after surgery (*n* = 394) was 19% (95% CI = 13–26) with I^2^ = 92.2%, according to the random effects model (Figure 2 and Figure 3).

**Figure 4:**
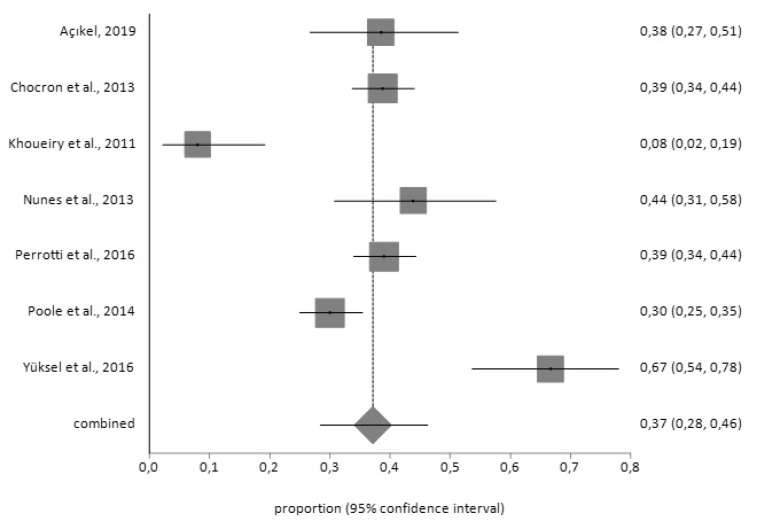
Forest plot for pre-CABG depression using Beck Depression Inventory (BDI).

**Figure 5:**
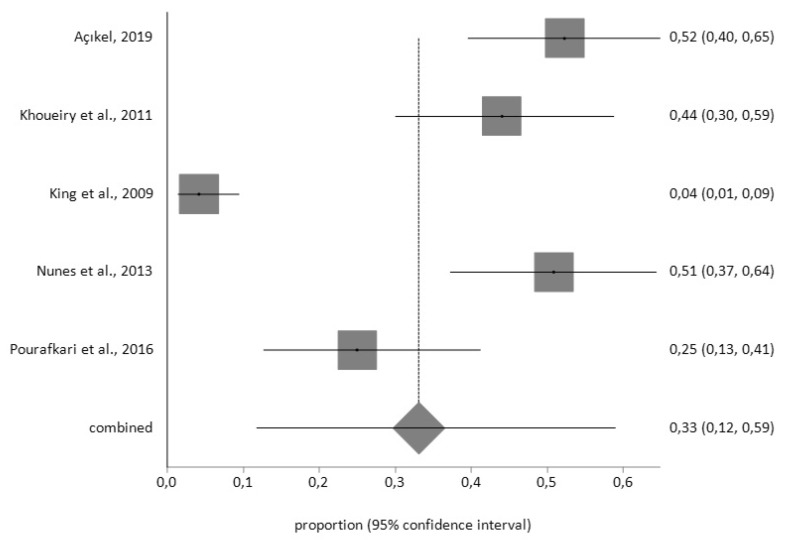
Forest plot for post-CABG depression using BDI.

For the BDI tool, the prevalence of depression prior to surgery (*n* = 469) was 37% (95% CI = 28–46) with a high degree of heterogeneity (I^2^ = 89.4%), while the prevalence afterwards (*n* = 97) was 33% (95% CI = 12–59) with a high degree of heterogeneity (I^2^ = 96.6%), according to the random effects model (Figure 4 and Figure 5).

**Figure 6:**
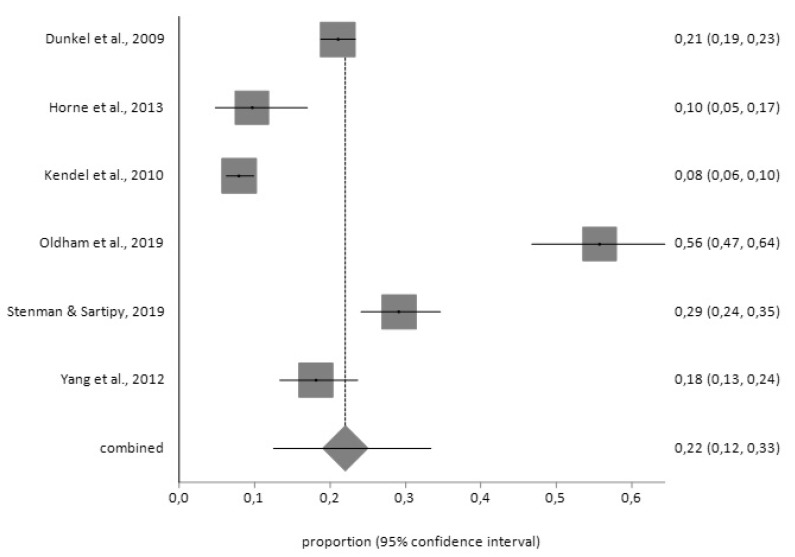
Forest plot for pre-CABG depression using Patient Health Questionnaire (PHQ-9).

**Figure 7:**
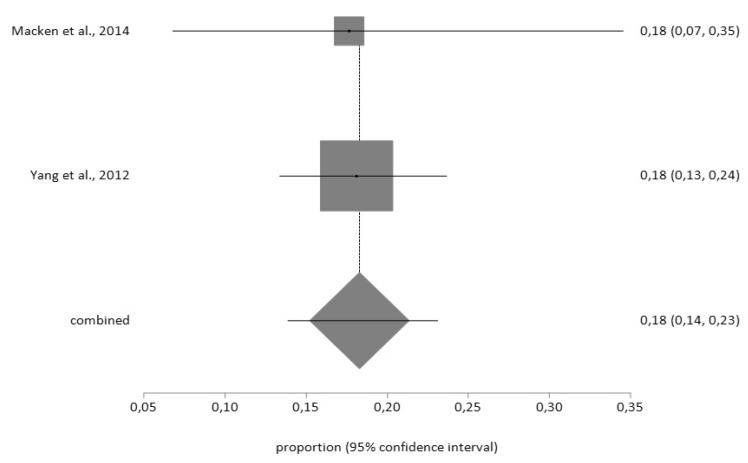
Forest plot for post-CABG depression using PHQ-9.

According to the PHQ-9 tool, the prevalence prior to surgery (*n* = 543) was 22% (95% CI = 12–33) with a high degree of heterogeneity (I^2^ = 97.5%) according to the random effects model; and, the prevalence of depression after surgery (*n* = 48), using the fixed effects model, was 18% (95% CI = 14–23) (Figure 6 and Figure 7), with a low degree of heterogeneity (I^2^ = 2%).

**Figure 8:**
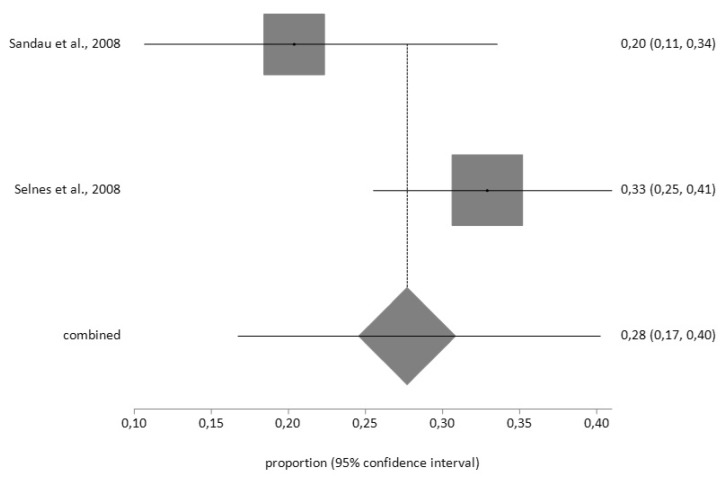
Forest plot for pre-CABG depression using Centre for Epidemiological Studies Depression Scale (CES-D).

**Figure 9:**
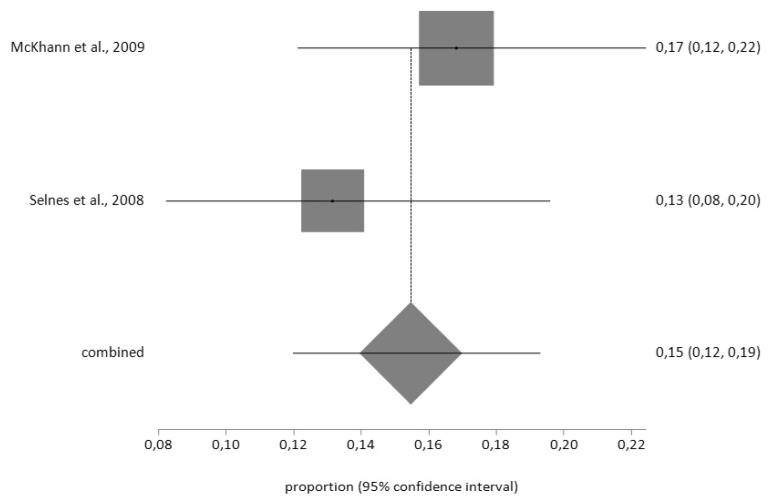
Forest plot for post-CABG depression using CES-D.

Finally, for CES-D, the prevalence of pre-CABG depression (*n* = 61) using the random effects model was 28% (95% CI = 17–40) with a moderate degree of heterogeneity (I^2^ = 66.9%); while the prevalence after surgery (*n* = 57) was 15% (95% CI = 12–19) with a low degree of heterogeneity (I^2^ = 2%), according to the fixed effects model (Figure 8 and Figure 9)

### 3.4. Levels of Depression Before and After CABG Surgery and Follow Up

Prior to CABG surgery, most of the authors report depression levels within the normal range, although others found mild [36,55,58,83,86] and moderate levels [27,35,66,69] (Table 1).

Post-surgery, most authors report normal levels, while others found mild [28,36,49,53,58,60,66,83,85,88], moderate [27,47,69], and severe [32] levels.

The majority of authors observed a positive impact on depression prevalence and levels after surgery, as well as in the short and medium term, although others found that these levels increased after surgery [28,32,33,48,49,55,57,89].

## 4. Discussion

The prevalence of depression obtained in this study varied between 19% and 37% prior to surgery, and between 15% and 33% after surgery, depending on the type of measurement tool used. Other studies that combine CABG with valve replacement have reported similar percentages, with depression prevalence ranging from 15% pre-CABG [90] to 37.7% post-CABG [51,91], associated with the development of the disease, worse quality of life, longer hospital stays, and high rates of hospital readmissions [8].

Normal levels of pre-CABG depression are observed, although other studies have indicated higher levels, from moderate to severe [92]. However, more than 25% of patients with normal levels are at risk of worsening, for which reason continuous reassessment can identify patients with transient symptoms of depression [93].

High levels of depression prior to the operation predict a worse quality of life [94,95], worse survival after a CABG [12,96], and more symptoms up to six months after surgery [97].

We have observed that depression levels did not go to remission, but they tend to improve in depressive symptoms, which is probably due to an improvement in the patient’s quality of life [98], and even due to greater optimism that facilitates commitment to adaptation [99]. Some authors have found a positive impact on patients from eight weeks [100], while others report a slight improvement from the first month post-CABG surgery [101]. For the majority of patients, depression persists after the surgery. Recent meta-analyses demonstrated that patients undergoing heart valve surgery are at risk of cognitive dysfunction up to six months after surgery [102,103].

Although there is a relationship between depression and CABG, its temporal onset is not clear. Depression can be a pre-existing condition, which increases the risk of cardiovascular disease that is related to behavioural alterations in diet, physical activity level, toxic habits, or poor adherence to treatment and recommendations [45]; or, can appear as a consequence of multiple postoperative complications, such as longer hospital stays [23], readmissions [104,105], general pain [104], or even when facing a series of lifestyle changes [12].

Without evaluation, it is unlikely that depression is being treated correctly. Some authors report that more than 50% of patients were receiving medical treatment for depression, even though they had no symptoms of depression [106]. For this reason, the use of measurement tools to confirm the presence and levels of depression makes it possible to identify the at-risk patients, and therefore carry out a more in-depth post-CABG follow-up, of at least nine months [93].

The current study highlights the importance of depression measures before and after CABG in assessing clinically meaningful mood disturbance, in order to provide early intervention. Systematic screening for depression in the period both before and after this procedure is crucial. Planned coaching combined with counselling can reduce these levels [36]. Cardiac rehabilitation programmes [107,108] and cognitive-behavioural therapies are also available, which reduce the levels of depression and even decrease the length of hospital stays [109]. However, further studies are needed to understand the potential prognostic implication of depression and investigate the best ways to approach the treatment of depression in this patient group.

Depression counselling prior to surgery can influence the post-surgical depression levels by positively improving a patient’s perception of illness control and management [13]. Planning is therefore an essential part of the healthcare process as it has the potential to promote self-care [36].

From a clinical perspective, these results suggest that strategies that are aimed to improve depression as a disorder, such as the application of policies and depression assessment protocols prior to CABG by health care providers, are essential, because the depression level might help risk stratification in patients undergoing CABG identifying the high-risk groups and the trajectory of recovery experienced [11].

This study has several limitations. Firstly, the heterogeneity in terms of prevalence is due to different estimation methods over time, differences in the timing of assessment and demographic differences between samples, different uses of cut-offs on questionnaire measures, as well as the use of various tools for assessing the symptoms of depression. Secondly, the measuring tools assess the severity of depression symptoms, but they do not replace a formal clinical diagnosis of depression.

## 5. Conclusions

There is a high presence of depression both before and after CABG surgery. While this study found an overall improvement in depressive symptoms after CABG, depression persists after the surgery for the majority of patients. The depression levels present prior to the operation may affect postoperative recovery.

Given the prevalence of depression and its impact, early detection is crucial, since it enables the identification of at-risk patients, through a clinical interview that uses validated measurement tools. This enables the medical team to implement preventive strategies as well as monitor the development of the depression.

## Figures and Tables

**Table 1:** Studies reporting prevalence and levels of coronary artery bypass graft surgery (CABG) depression.

Study CABG (First, Elective, Emergency)	Design and Sample	Depression Screening Instrument	Timing of Assessment	M (SD)/Prevalence		Main Results	EL/RG
				Pre	Post		
Abbott et al., [26] USA Elective CABG	RCT *n* = 226 83% male	HADS	After CABG	–	Cluster 1: 5.9 (4.3) Cluster 2: 8.2 (4.8) Cluster 3: 11.8 (6.9)	Elderly patients with more symptoms and chronic illnesses are more prone to depression	1a/A
Aburuz, [27] Jordan Elective CABG	Cohort *n* = 227 78% male	HADS	2-weeks before, 1-month after	12.76 (6.80) Normal: 57.26% Mild: 11.90% Moderate-severe: 30.84%	11.11 (6.78) Normal: 59.47% Mild: 13.66% Moderate-severe: 26.87%	Pre-operative depressive symptoms increased postoperative hospital length of stay	2c/B
Açıkel, [28] Turkey Elective CABG	Cohort *n* = 65 76.9% male	BDI	1-day before 3–7 days, 1-month after	8.12 (5.44) Normal: 61.5% Mild: 30.8% Moderate: 7.7% Severe: 0%	**3rd day:** 12.43 (6.36) Normal: 35.4% Mild: 40.0% Moderate: 23.1% Severe: 1.5% **7th day:** 11.66 (6.95) Normal: 40.0% Mild: 30.8% Moderate: 27.7% Severe: 1.5% **1 month:** 12.29 (9.08) Normal: 47.7% Mild: 26.2% Moderate: 23.1% Severe: 3.1%	Depression levels increase during postoperative CABG period	2c/B
Afridi et al., [29] Pakistan First-time CABG	Cohort *n* = 134 84.3% male	HAM-D	2 days before, at discharge, 6-months follow-up	98.5% Mild: 71.6% Moderate: 23.9% Severe: 1.5% Very severe: 1.5%	**At discharge:** 80.6% Mild: 73.9% Moderate: 2.23% Severe: 2.23% Very severe: 2.23% **6-months:** 16.4%	Depression is commonly reported before and after CABG and influences the quality of life of the patients	2c/B
Ajtahed et al., [30] Iran First-time CABG	RCT *n* = 75 67% male	DASS	After CABG	–	**Control group:** **Group 1:** Normal: 60% Mild: 40% Moderate: 24% Severe: 12% Extremely severe: 0% **Group 2:** Normal: 52% Mild: 8% Moderate: 32% Severe: 0% Extremely severe: 8% **Group 3:** Normal: 45% Mild: 9.1% Moderate: 13.6% Severe: 13.6% Extremely severe: 18.2%	Training cognitive rehabilitation can improve cognitive functions and quality of life in patients after CABG surgery	1a/A
Ammouri et al., [31] Jordan First-time CABG	Cross-sectional *n* = 100 80% male	CSS	2-weeks after discharge	–	3%	Pain, leg swelling, poor appetite and trouble sleeping are the most frequent symptoms after CABG	2c/B
Amouzeshi et al., [32] Iran Elective CABG	Cross-sectional *n* = 54 68% male	BDI	1 day before and after ICU	11.7 (7) Minimal: 55.4% Mild: 28.6% Moderate: 16.1% Severe: 0%	**Male:** 31.5 (10.60) **Female:** 29.3 (10.55) Minimal: 0% Mild: 17.9% Moderate: 32.1% Severe: 46.4%	No relationship between age, sex, marital status, and education level with post-operative depression	2c/B
Azzopardi & Lee, [33] Australia Elective CABG	Cohort *n* = 48 85.4% male	BDI	Before, 6-weeks after, 1–2 years follow-up	7.31 (4.1)	**2 years:** 7.90 (7.1)	Depression levels 2 years after CABG were not severe	2b/B
Bay et al., [34] USA Elective CABG	RCT *n* = 170 75% male	HADS	Baseline,1–6 months after	**Control group:** 7.3 (3.7)	**Control group:** **1-month:** 3.0 (3.1) **6-months:** 3.0 (3.1)	A coping religious intervention can reduce depression levels up to 6 months after surgery	1a/A
Beresnevaite et al., [35] Lithuania Elective CABG	Cross-sectional *n* = 109 100% male	SCL-90R	1-day before after	63.13 (8.22) High level: 23%	–	Preoperative depression score is related with a length stay hospital (p < 0.001) and perioperative complications (*p* < 0.05)	2b/B
Cebeci & Çelik, [36] Turkey First-time CABG	Quasi-experimental *n* = 52 80.8% male	HADS	1-day before, 1-day,1-week, 1-month after	8.3 (3.6)	**At discharge:** 7.9 (4.2) **1-week:** 8.2 (4.5) **1-month:** 7.7 (4.3)	At the time of admission, patients had a higher level of depression than at the time of discharge	1b/A
Chocron et al., [37] France First-time CABG	RCT *n* = 361	BDI	Before CABG	39%	–	Antidepressant treatment did not affect the morbidity and mortality events after CABG surgery	1a/A
Colella et al., [38] Canada First-time CABG	RCT *n* = 124 100% male	BDI	At discharge, 6–12 weeks after	–	**Control group:** **After:** 8.87 (4.74) **6-weeks:** 5.84 (5.30) **12-weeks:** 4.43 (5.26)	Physiological and psychological challenges after CABG increases the depression risk	1a/A
Dal Boni et al., [39] Brazil Elective CABG	Cross-sectional *n* = 78 67% male	BDI	Before, 2-months after	8.49 (6.87)	5.01 (6.61)	CABG had a positive impact on the patient’s quality of life	2b/B
Doering et al., [40] USA First-time CABG	Cohort *n* = 67 100% female	HAM-D	At discharge, 1 month after	–	41.79%	Six months after CABG, women with major depression have at increased risk for infections	2b/B
Donohue et al., [41] USA Elective CABG	RCT *n* = 2485	PHQ-2	At discharge	–	56%	A nurse-guided intervention in the mental health area reduces the level of depression and health costs post-CABG	1a/A
Dunkel et al., [42] Germany Elective CABG	Cross-sectional *n* = 1238 72% male	PHQ-9	Before CABG	21.6%	–	Lower age, female gender, less than 10 years of education and living alone are related to depression symptoms	2b/B
Dunkel et al., [43] Germany Elective CABG	Cross-sectional *n* = 971 80.1% male	PHQ-9	1–3 days before, 1 year after	5.61 (4.31)	–	Female gender is related to depression symptoms Attributions to stress, personality and destiny are associated with higher depression scores	2b/B
El-Baz et al., [44] Netherlands and Slovakia Elective CABG	Observational multicentre *n* total = 226 *n*1 = 114 Slovakia *n*2 = 112 Netherlands 80% male	HADS	Before CABG	*n*1 = 5.01 (3.73) *n*2 = 4.96 (3.16)	–	Female gender, smoking, lower education, and lower social support are risks factors of depression	2b/B
Elliott et al., [45] Australia Elective CABG	Cohort *n* = 174 80% male	POMS-D	Before, 2–6 months after	10.50 (11.76)	**2-months:** 7.38 (9.41) **6-months:** 8.32 (10.52)	The young, male and smoking are the main risk factors of depression	2b/B
Feuchtinger et al., [46] Germany First-time CABG	Cross-sectional *n* = 24 37.5% male	HADS	1-day before	6.7 (5.1) Low: 54.17% Moderate: 20.83% Severe: 25%	–	Interventions such as information, spiritual support or cognitive behavioral therapy are the key to reduce the feeling of fear before CABG surgery	2b/B
Freedland et al., [47] USA Elective CABG	RCT *n* = 123 50% male	BDI HAM-D	1 year after	–	BDI = 22.26 (1.3) HAM-D = 19.53 (1)	Improvement in perceived cognitive impairment correlated with improvement in depression	1a/A
Gallagher & McKinley, [48] Australia Elective CABG	Cohort *n* = 155 74% male	HADS	Before, after surgery, 2-weeks after	4.10 (3.22) 16%	**After:** 18.2% 4.67 (3.49) **2-weeks:** 45% 6.58 (4.03)	26.5% of patients reported low perceptions of control before CABG, 22% after surgery and 10.3% at discharge	2b/B
Gelogahi et al., [49] Iran Elective CABG	RCT *n* = 40 37.5% male	DASS	Before, after	6.67 (4.7)	12.1 (8.1)	Nurses interventions can reduce depression levels after surgery	1a/A
Hazavei et al., [50] Iran First-time CABG	Quasi-experimental *n* = 27 77.8% male	CDS	Before, 4-8 weeks after	104.5 (30.4)	**2-months after:** 89.2 (27.8)	Most patients lacked the skills in health education and lifestyle-related with coronary artery disease	1b/A
Horne et al., [51] Canada Elective CABG	Cohort *n* = 104	PHQ-9	Before	60.6%	–	Length of stay (more than 7 days) is associated with a higher risk of depression	2b/B
Hweidi et al., [52] Jordan Elective CABG	Cross-sectional *n* = 143 53.1% male	SDS	2 days after	–	Mild: 32.2% Moderate: 60.1% Severe: 5.6%	Depression is related to female, unmarried and unemployed patients	2b/B
Kendel et al., [53] Germany Elective CABG	Cohort *n* = 351 77% male	PHQ-9	2 months, 2 years after	–	Male: 5.38 (4.2) Female: 6.84 (4.8)	Females have a higher level of depressive symptoms	2b/B
Kendel et al., [54] Germany Elective CABG	Cohort *n* = 883 80.2% male	PHQ-9	1–3 days before	5.38 (4.09) 8.5%	–	Depression is related to a deterioration of physical condition in patients undergoing CABG surgery	2b/B
Khoueiry et al., [55] USA Elective CABG	Cohort *n* = 50 56% male	BDI	Before, 1–3–6–9 months follow-up	8%	**After:** 60% **3-months:** 44% **6-months:** 40% **9-months:** 44%	Age and gender are not correlated with depression levels	2b/B
King et al., [56] Canada First-time CABG	Cohort *n* = 120 100% male	BDI CDS	At discharge, 6–12–36 weeks follow-up	–	**BDI** **At discharge:** 8.08 (4.76) 4.3% **6-weeks:** 5.82 (5.36) 1.9% **12-weeks:** 4.81 (4.73) 1.9% **36-weeks:** 4.31 (5.81) 2.1% **CDS** **At discharge:** 74.46 (24.29) 17.2% **6-weeks:** 59.58 (25.19) 7.6% **12-weeks:** 54.56 (23.06) 6.7% **36 weeks:** 51.22 (23.17) 4.3%	Family reduces the risk of depression	2b/B
Korbmacher et al., [57] Germany Elective CABG	Cohort *n* = 135 77% male	HADS	1–2 days before,1-week, 6-months after	4.3 (3.1) 20.7%	**1-week:** 5 (3.9) 24% **6-months:** 4.7 (4.3) 28%	Hight levels of depression are not associated with mortality. A 24.2% of patients with normal scores before surgery suffers depression 6-months latter	2b/B
Kozora et al., [58] USA Elective CABG	Cohort *n* = 1156 99.2% male	BDI	After, 1-year follow-up	9.9 (7.65)	8.9 (7.85)	Older age and lower education are related to depression levels	2b/B
Macken et al., [59] USA Elective CABG	Quasi-experimental *n* = 34 76.5% male	PHQ-9	After CABG	–	Control group: 18%	An intervention cardiac program can reduce depressive symptoms	1b/A
McGrady et al., [60] USA Elective CABG	Quasi-experimental *n* = 91	BDI	After CABG	–	9.2 (7.5)	The symptoms can affect adherence to prescribed treatment and may also affect morbidity and mortality	1b/A
McKenzie et al., [61] UK Elective or emergency CABG	Cross-sectional *n* = 111 82.9% male	HADS	After CABG	–	3.16 (3.61)13.5%	Post-operative depression predicts activities of daily living functioning	2b/B
McKhann et al., [24] USA Elective CABG	Cohort *n* = 220 73.6% male	CES-D	After, 3 months, 1–3–6 years after	–	**Baseline:** 13.2 (9.8) 32.4% **3-months:** 10.2 (9.9) 24.1% **1 year:** 9.1 (9.8) 17.3% **3 year:** 8.9 (9.5) 11.8% **6 year:** 10.1 (9.4) 16.8%	Depressed patients tended to have more memory complaints	2b/B
Modica et al., [62] Italy Elective CABG	Cross-sectional *n* = 1179 80% male	HADS	After CABG	–	Moderate-severe: 10.4% Male: 9.2% Female: 15.4%	Female gender is related to a higher depression score	2b/B
Moser et al., [63] USA Elective CABG	Observational multicentre *n* = 131 94% male	MAACL	After CABG	–	13.0 (5.5) 53%	Factors such as being a woman and have lower educational attainment are related to depression	2b/B
Murphy et al., [64] Australia Elective CABG	Cohort *n* = 184 79% male	HADS	Before, 2–6 months follow-up	5.35 (4.01)	**2-months:** 4.16 (3.71) **6-months:** 3.87 (3.51)	Over 6-months follow-up patients show a minor score of depression	2b/B
Nair et al., [65] India Elective CABG	Quasi-experimental *n* = 500 20.2% male	HADS	6 months after	–	20.2%	11.6% of patients after CABG adhered to healthy lifestyle practices	1b/A
Nemati & Astaneh, [66] Iran Elective CABG	Cohort *n* = 71 73% male	HADS	Before, 4-weeks after	Male: 13.58 (8.54) Female: 17.88 (7.54)	Male: 9.51 (6.00) Female: 15.05 (8.63)	CABG surgery can decrease the level of depression in a short-term follow-up	2b/B
Nunes et al., [67] Brazil Elective CABG	Cohort *n* = 57 68.42% male	BDI	Before, 6-months after	Minimal: 56.14% Mild: 26.32% Moderate: 12.28% Severe: 5.26%	Minimal: 49.12% Mild: 29.82% Moderate: 17.54% Severe: 3.51%	Improvement the quality of life with CABG surgery reducing depressive symptoms	2b/B
Okamoto et al., [68] Japan Elective or emergency CABG	Cross-sectional *n* = 79 75.9% male	HADS	1–5 years after	–	Mild: 10.1% Moderate-severe: 10.1%	Depression in CABG patients is related to a decrease in functional status or activities of daily living	2b/B
Oldham et al., [69] USA First-time CABG	Cohort *n* = 131 73% male	HAM-D PHQ-9 GDS	Before	HAM-D: 9.9% 16.3 (5.4) PHQ-9: 56.2% 13.4 (3.9) GDS: 6.9 (3.6)	–	Preoperative depression predicts post-CABG cognitive health	2b/B
Perrotti et al., [70] France Elective CABG	RCT *n* = 359 85% male	BDI	Before,1 year after	39.6%	–	In the first year after CABG, depressed patients have a lower improvement and quality of life	1a/A
Perrotti et al., [71] France Elective CABG	Cohort *n* = 272 78% male	HADS	2-weeks before	6%	–	CABG surgery improve the functional mobility, quality of life and maintenance of an independent status	2b/B
Phillips-Bute et al., [72] USA Elective CABG	Cohort *n* = 427 70% male	CES-D	Before, 6 months, 1 year after	Mild-Severe Male: 28% Female: 57%	Mild-severe: **Male:** 6-months: 17% 1-year: 17% **Female:** 6-months: 33% 1-year: 32%	Depressed patients are more prone than nondepressed patients to have a new cardiac event within 2 years of CABG	2b/B
Poole et al., [23] UK First-time CABG	Cohort *n* = 310 86% male	BDI	29 days before, after surgery	8.68 (6.61) 30.3% Minimal: 69.7% Mild: 25.5% Moderate-severe: 4.8%	8.33	Pre-operative depression is associated with longer postoperative hospital stays. The young, female gender, overweight, smoking and hypertension variables are related to depression symptoms	2b/B
Pourafkari et al., [73] Iran Elective CABG	Quasi-experimental *n* = 40 82% male	BDI	After CABG	–	25% Minimal: 75% Mild: 12% Moderate: 8% Severe: 5%	The emergence of new-onset depression after CABG is associated with a poor outcome	1b/A
Rezaei et al., [74] Iran Elective CABG	Cohort *n* = 135 75% male	SCL-90R	6 months after	–	1.17 (0.75) 44.22%	The prevalent mental disorder after CABG is depression followed by sensitivity, paranoia, hostility, anxiety, obsession, somatization, phobia, and psychosis	2b/B
Sandau et al., [75] USA Elective CABG	Cohort *n* = 54 78% male	CES-D	Before, 3-months after	14.2 (8.6) 20%	10.4 (7.5)	Depressive symptoms remain constant from pre- to postoperatively at 3 months	2b/B
Schwarz et al., [76] Germany Elective CABG	Cohort *n* = 47 89% male	HADS	Before, 3-months after	5.0 (3.4)	3.8 (3.1)	Depression and health-related quality of life are not associated with cognitive dysfunction after CABG	2b/B
Selnes et al., [25] USA Elective or emergency CABG	Cohort *n* = 152 76% male	CES-D	Before, 12–72 months follow-up	13.2 (9.6) 33%	9.5 (9.2) 13%	CABG patients had a decline of score 72-months after	2b/B
Sorensen & Wang, [77] USA First-time CABG	Cohort *n* = 70 66% male	GDS	Before, 6-weeks after	3.1 (2.5) 24.2%	2.4 (2.3) 15.9%	Women had greater depression pre-operative and post-operative. Length of stay and age are not related to depression	2b/B
Spezzaferri et al., [78] Italy Elective CABG	Cohort *n* = 118 100% male	CBA 2.0-D	At discharge, 1 year after	–	**At discharge:** 12.7% **1 year:** 5.9%	1 year after CABG depression level decreased	2b/B
Stenman & Sartipy, [79] Sweden Elective and emergency CABG	Cohort *n* = 302	PHQ-9	Before	29%	–	Depressive symptoms are twice as frequent in women as in men	2b/B
Thomas et al., [80] India First-time CABG	Quasi-experimental *n* = 100 85% male	HADS	Before, 1-week, 1 month after	4.10 (3.30)	**1-week:** 2.03 (2.60) **1-month:** 1.26 (1.82)	Medical adherence behavior is related to depression six weeks after surgery	1b/A
Tsai et al., [81] Taiwan First-time CABG	Cohort *n* = 198 81% male	CSS	Before, 1–6 weeks, 3 months follow-up	2.42 (2.64)	**1-week:** 1.41 (2.00) **6-weeks:** 1.24 (1.86) **3-months:** 0.96 (1.70)	Age, a longer stay in ICU, smoking, and lack of exercise are related to worse symptoms. 88% of patients have a trajectory of depression levels that decrease over time	2b/B
Tully et al., [82] Australia First-time CABG	Cohort *n* = 226 83% male	DASS	Before, 4 days after	20.1%	23.5%	Readmission is related to a higher depression score. Depression symptoms are associated with morbidity	2b/B
Tully et al., [83] Australia First-time CABG	Cohort *n* = 226 83% male	BDI	Before, after surgery	8.62 (6.23)	9.05 (6.40)	Pessimism, past failure, self-criticalness and, worthlessness are associated with cardiac morbidity and mortality	2b/B
Yang et al., [84] China First-time CABG	Cohort *n* = 232 81% male	PHQ-9	3-days before, 6-months after	4.8 (5.0) 18.1%	4.2 (5.0) 18.1%	Preoperative depression is associated with women gender	2b/B
Yang et al., [85] Taiwan Elective and emergency CABG	Cross-sectional *n* = 87 74.7% male	HADS	1 week, 1 month after	–	**1 week:** Mild: 17.2% Moderate-severe: 60.9% **1 month:** 8.75 (4.63) Mild: 24.1% Moderate-severe: 35.6%	Depression is related to sleep quality after CABG surgery	2b/B
Yüksel et al., [86] Turkey Elective and first-CABG	Cohort *n* = 63 G1: diagnosed after experiencing an ACS G2: diagnosed without an ACS	BDI	Before	G1:14.9 (9.5) G2: 12.1 (7.4) 66.6% Mild: 22.2% Moderate-severe: 44.4%	–	Patients in both groups were found to be depressed and hopeless about the future	2b/B
Zimmerman et al., [87] USA Elective CABG	RCT *n* = 226 83% male	CSS	At discharge	–	19%	Health care providers must assist the patients before hospital discharge to identify the risks and difficulties in patients after CABG up to 6 months after surgery	1a/A

ACS = Acute Coronary Syndrome; BDI = Beck Depression Inventory; CABG = Coronary Artery Bypass Graft; CBA 2.0-D = Depression scales of the Cognitive Behavioural Assessment; CDS = Cardiac Depression Scale; CES-D = Center for Epidemiological Studies Depression Scale; CSS = Cardiac Symptom Survey; DASS = Depression, Anxiety, Stress scale; GDS = Geriatric Depression Scale; HADS = Hospital Anxiety and Depression Scale; HAM-D = Hamilton Rating Scale for Depression; ICU = Intensive care unit; MAACL = Multiple Affect Adjective Checklist; PHQ-2 = Patient Health Questionnaire 2-item; PHQ-9 = Patient Health Questionnaire 9-item; POMS-D = Profile of Mood State Depression Scale; RCT = Randomized Clinical Trial; SDS = Self-rating Depression Scale; SCL-90R = Symptom Checklist-90 Revised.

## Supplementary Materials

The following are available online at https://www.mdpi.com/2077-0383/9/4/909/s1, Table S1: Depression Assessment Instruments Used by the 65 Studies Included in the Systematic Review [110,111,112,113,114,115,116,117,118,119,120,121,122].
