# Supplementary material for: Prevalence of Depression in Coronary Artery Bypass Surgery: A Systematic Review and Meta-Analysis
Source: J Clin Med. 2020 Mar 26;9(4):909. doi: 10.3390/jcm9040909 (PMC7230184; doi:10.3390/jcm9040909)
Supplement: Supplementary file 1 [file jcm-09-00909-s001.pdf]

Table S1. Depression Assessment Instruments Used by the 65 Studies Included in the Systematic Review

**Depression instrument**	**Studies**	**Range score**	**Score**
Beck Depression Inventory (BDI) [9]	N = 17	0–63	0–9: normal<br>10–16: mild depression<br>17–29: moderate depression<br>30–63: severe depression<br>Cut-off score for depression ≥10
Cardiac Depression Scale (CDS) [110]	N = 2	26–182	Higher scores indicate higher depression levels
Center for Epidemiological Studies Depression Scale (CES-D) [111]	N = 4	0–60	<16: normal<br>16–26: mild depression<br>≥ 27: severe depression<br>Cut-off score for depression > 16
Cardiac Symptom Survey (CSS) [112]	N = 3	0–10	Higher scores indicate higher depression levels
Depression, Anxiety, Stress Scale (DASS) (depression subscale) [113]	N = 3	0–42	0–9: normal<br>10–13: mild<br>14–20: moderate<br>21–27: severe<br>>28: extremely severe
Depression scales of the Cognitive Behavioural Assessment (CBA 2.0-D)[114]	N = 1	-	Higher scores indicate higher depression levels
Geriatric Depression Scale (GDS) [115]	N = 2	0–30	0–9: normal<br>10–19: mild depression<br>20–30: severe depression
Hamilton Rating Scale for Depression (HAM-D)[116]	N = 4	0–52	0–7: normal<br>8–16: mild depression<br>17–23: moderate depression<br>>24: severe depression

Hospital Anxiety and Depression Scale (HADS) (depression subscale) [10]	N = 18	0–21	0–7: normal<br>8–10: mild<br>11–14: moderate<br>15–21: severe<br>Cut-off score for depression ≥ 8
Multiple Affect Adjective Checklist Depression Scale (MAACL)[117]	N = 1	0–40	Higher scores indicate higher depression levels
Patient Health Questionnaire (PHQ-2) [118]	N = 1	0–6	Higher scores indicate higher depression levels
Patient Health Questionnaire 9-item (PHQ-9) [119]	N = 9	0–27	0–4: minimal<br>5–9: mild<br>10–14: moderate<br>15–19: moderate-severe<br>20–27: severe<br>Cut-off score for depression >10
Profile of Mood States Depression Scale (POMS-D) [120]	N = 1	0–60	Higher scores indicate higher depression levels
Self-rating Depression Scale (SDS) [121]	N = 1	20–80	25–49: normal<br>50–59: mild depression<br>60–69: moderate depression<br>>70: severe depression
Symptom Checklist-90 Revised (SCL-90R) [122]	N = 2	-	Higher scores indicate higher depression levels
